# Supplementary material for: Cargo adaptors use a handhold mechanism to engage with myosin V for organelle transport
Source: bioRxiv. 2025 Mar 25:2025.03.24.645041. Preprint. [Version 1] doi: 10.1101/2025.03.24.645041 (PMC11974856; doi:10.1101/2025.03.24.645041)
Supplement: Supplement 1 [file media-1.pdf]

Supplement

Table S1 shows Vac17 suppressor mutant candidates identified from the PCR screen. The percentage of cells containing an inherited vacuole in the bud was measured. For each group, between 30-200 cells were analyzed, with data collected from at least two biological replicates. For more specific details about the screen, see (Ishikawa et al., 2003). **Table S2** lists the primers used for this study. **Table S3** lists strains and **Table S4** lists plasmids used in this study. **Table S5** contains information regarding cryo-EM data collection, refinement and statistics. **Fig. S1** shows that the *vac17ΔH* mutant is defective in its localization to sites of polarized growth, but not with Vac8, and that Vac17(H) binds directly to the Myo2 tail *in vitro*. **Fig. S2** shows that the expression of Vac17 in the *vac17ΔH* mutant is elevated, and that the vacuole inheritance in small buds is delayed in the mutant. **Fig. S3** shows an overview of the cryo-EM specimen's purification, data collection. refinement and validation statistics. **Fig. S4** shows that Vac17 and Mmr1 bind overlapping sites on Myo2. **Fig. S5** shows cryo-EM processing workflow and validation statistics for the composite map of Vac17 bound to Myo2 at two sites.

Figure Legends

Supplementary Figure 1. the *vac17ΔH* mutation specifically impairs its interaction with Myo2.

(A) In wild-type cells, Vac17 accumulates on the vacuole surface closest to the bud cortex, while Myo2 localizes to either the bud tip or the mother-bud neck. Upon vacuole

arrival at the bud tip, Vac17 undergoes degradation. In the *myo2(D1297N)* mutant, which is defective in vacuole inheritance, Vac17 is distributed around the vacuole, whereas *myo2(D1297N)* maintains its normal localization at sites of polarized growth. The *vac17ΔH* and *myo2(D1297N)* double mutant does not further alter the localization pattern of Vac17 or Myo2 compared to the *myo2(D1297N)* mutant. Scale bars = 5 μm.

(B) *vac17ΔH* still colocalizes with Vac8-RFP (pseudo-colored yellow), indicating that its binding to Vac8 is not impaired.

(C) In the absence of Vac8, Vac17 localizes presumably with Myo2, at the bud tip or the mother-bud neck, but *vac17ΔH* is cytosolic. Scale bars = 5 μm.

(D) Deletion of the canonical Vac17(MBD) abolishes vacuole inheritance.

(E) (Top) SDS-PAGE analysis of apo Myo2 tail purification. The peak elutes at 15.5 -16 ml. (Middle) Analysis of the Myo2 tail+Vac17(MBD) complex, showing an expected peak between 15.5 - 16 ml. There is also a higher molecular weight complex with both Vac17 and Myo2 at 13.5 - 14 ml. This latter peak was not analyzed further. (Bottom) Immunoblot analysis of the gel filtration experiment from (**Fig. 1 E**; top panel, lighter blue trace) shows the Myo2 tail+Vac17(H) complex at 14.5 - 15 ml. The membrane used to detect Myo2 was stripped and re-probed for MBP. The arrowheads correspond with the same arrowheads shown in (**Fig. 1 E)**: Gray = apo Myo2 tail; orange = free MBP; blue = Myo2 tail+Vac17(H); green = Myo2 tail+Vac17(MBD).

Supplementary Figure 2. Elevated levels of Vac17 in the *vac17ΔH* mutant may eventually suppress the vacuole inheritance defect.

(A-C) Buds that ultimately received vacuoles were chosen from (**Fig. 2, A and C)** and analyzed retrospectively.

(A, B) Vacuole inheritance over time following bud emergence (n=24 cells for wild-type; n=7 for *vac17ΔH* mutants). Time 0 represents cells where vacuole inheritance occurred concomitantly with bud emergence. After time 0, all cells that achieved vacuole inheritance at any time were included.

(C) Analysis of the timing of vacuole inheritance in large buds of *vac17ΔH* mutant cells that lacked a vacuole at the start of imaging (n=13). Large buds inherited vacuoles at times ranging from 20 seconds to 40 minutes.

(D) Western blot analysis of lysates from cells expressing Vac17 from a CEN (low copy) plasmid, compared to vector alone (n=3). Cells were harvested during mid-log phase (OD_{600} 0.4-0.6). The vector control did not contain detectable bands. This confirms that the doublet bands in Vac17-3xEnvy and *vac17ΔH-3xEnvy* are Vac17 protein. Both bands were included in the quantification. Deletion of the PEST motif, which is essential for Vac17 degradation, results in elevated Vac17 levels.

Supplementary Figure 3. Purification and cryo-EM analysis of the Vac17-Myo2 complex.

(A) Example of preparative gel filtration showing co-expression of recombinant Vac17(H+MBD) and Myo2 tail. The black arrowhead indicates the complex formation for Vac17(H+MBD) and Myo2, which was sampled for data collection (top). The orange arrowhead marks free MBP, and the gray arrowhead indicates apo Myo2 tail. SDS-PAGE analysis of fractions from volumes 10-18 ml is shown.

(B) Example micrograph with selected particles. Scale bar = 100 nm.

(C) 2D class averages of particles processed using CryoSPARC. Scale bar = 130 Å.

(D) Local resolution range of the reconstruction.

(E) Model statistics (top) and Euler distribution of particles.

(F) Comparison of the Myo2 tail (2F6H; yellow) with the AlphaFold model, trimmed to fit the cryo-EM reconstruction (gray). Black circles indicate regions resolved in the cryo-EM map. Red circle highlights a predicted region lacking empirical data.

Supplementary Figure 4. Mmr1 and Vac17(MBD) bind a shared site on Myo2 and are anti-parallel to each other.

(A) AlphaFold model docked within the cryo-EM map of Vac17(MBD; 127-147) bound to Myo2 tail. Vac17-binding region (blue), Mmr1-binding region (red) and overlapping region of Vac17 and Mmr1 on Myo2 (pink) (Eves et al., 2012; this study).

(B) Vac17(MBD) extends along a hydrophobic groove (gold) on the Myo2 tail, which contains multiple surface residues critical for vacuole inheritance.

(C) Vac17(L137) makes hydrophobic interactions with Myo2(L1301 and L1229).

(D) An Mmr1 peptide(408-425) bound to the Myo2 tail (PDB: 6IXP) was docked into the EM reconstruction. The Mmr1 peptide partially fits into the density of Vac17(MBD), consistent with a previous study showing that Vac17 and Mmr1 peptides compete for access to Myo2 *in vitro* (Eves et al., 2012).

(E) Both the Vac17 and Mmr1 adaptor proteins contain serine phosphosites (depicted as space-filing model) that are important for dissociation from Myo2 (Wong et al., 2020; Obara et al., 2022).

Supplementary Figure 5. Vac17-Myo2 cryo-EM data processing workflow.

Table S1 Vac17 suppressor screen

% Vacuole inheritance		
VAC17 Suppressor candidates		
	wild-type Myo2	*myo2-N1304S*
I15V	96	48
I28R	94	41
N53Y	95	50
Y55H	95	51
S57F	93	60
N60Y	94	58
N60I	98	49
L96M	97	57
T110P	93	66
R126S	95	51
I140V	95	52

Table S2 Select primers used in this study

	***In vitro* studies**	
LHP211	F SalI - vac17(1-109) - PstI	tcagacGTCGACATGGCTACGCAAGCCCTGGAAGAC
LHP212	R SalI - vac17(1-109) - PstI	tcagacCTGCAGTTATGCCAGTTCATTCAGGCGCAGGGT
LHP216	F SalI - vac17(110-157) - PstI	tcagacGTCGACACGGTGCCGAATGAAGCTAGTAACG
LHP217	R SalI - vac17(157) - PstI	tcagacCTGCAGTTAGGATTTTTCGGCGGTTTACTCGGGG
LHP364	myo2 tail-V1448A	GCTACTGCAAGcCCGTAAGTATAC
LHP377	myo2 tail-R1449E	GCTACTGCAAGTCgaaAAGTATACTATCGAAGAC
LHP378	myo2 tail-K1408A	TAATTTCTTGTCGTGGgcAAGGGGTCTT
LHP380	myo2 tail-E1338R	CGCGGGAcgAACCAGCGGGTTTTT
	***In vivo* studies**	
LHP319	myo2-W1407Y	CGTAATTTCTTGTCGTacAAAAGGGGTCTTC
LHP285	myo2-E1222K	GGCTGACCAAGCAAAGTaAAAGCTTTCTTGCCC
LHP275	myo2-V1448A	CCGCTAAGCTACTGCAAGcCCGTAAGTATACTATCG
LHP277	myo2-R1449E	GACCGCTAAGCTACTGCAAGTCgaaAAGTATACTATCGAAGAC
LHP280	myo2-I1462R	CGAAGACATTGATATCTTAAGAGGAAgaTGTTATTCGCTAACACCTGCAC
KBP1	myo2-F1264E	CTCATTTGTGGTGgaaGCTCTAAACTCTATTTTAACCG
KBP2	myo2-F1275E	CCGAAGAAACGgaaAAAAACGGCATGA
LHP352	myo2-L1411W	GTCGTGGAAAAGGGGTtggCAATTGAACTACAACG
LHP353	myo2-T1418Q	GGGGTCTTCAATTGAACTACAACGTTcaaAGATTAGAGGAATGG
LHP356	myo2-I1458A	CGAAGACATTGATgcCTTAAGAGGAATTTGTTATTCGC
LHP357	myo2-E1484R	GGTGGCAGACTATagGTCTCCAATTCCAC
LHP344	myo2-Y1483A	AGGTGGCAGACgcTGAGTCTCCAA
LHP347	myo2-K1408A	GTAATTTCTTGTCGTGGgcAAGGGGTCTTC
LHP348	myo2-E1338R	TTTCAGCGCGGGAcgAACCAGCGGGTTTT

LHP325	vac17-F132Y	ccagaagtagtttagggtcatAtcaacctcgacc
LHP326	vac17-L137A	catttcaacctcgaccaGCgaaaataattgagaggc
LHP328	vac17-R142E	ccattgaaaataattgagGAgcaacgtctgtgtatggtaactcc
LHP253	vac17-K138E	gtcatttcaacctcgaccattgGAaataattgagaggcaacg
LHP255	vac17-R135E	gggtcatttcaacctGAaccattgaaaataattgagaggc
**Internal deletions of Vac17**		
LHP339	Q5SDM_12/2/2022_R 110-	GGCCAGCTCGTTCAACCT
LHP340	Q5SDM_12/2/2022_F -157	GTAGGCTTTAACCCCATCAATG
LHP107	Q5SDM_HHPRED pRS416-vac17(18-108 del)-3XENVY_F	GCCACCGTCCCAAATGAA
LHP108	Q5SDM_HHPRED pRS416-vac17(18-108 del)-3XENVY_R	CGACCTTATTAAAAGCCTCTC

Table S3 Strains used in this study

Strain	Genotype	Reference
LSW5798	*MATa*, *ura3-52*, *leu2-3,-112*, *his3-Δ200*, *trp1-Δ901*, *lys2-801*, *suc2-Δ9*, *vac17Δ::TRP1*	Tang *et al*., 2003
LWS8195	*MATα, ura3-52, leu2-3,-112, his3-Δ200, trp1-Δ901, lys2-801, suc2-Δ9, pep4-Δ1137, vac17Δ::TRP1, myo2Δ::TRP1, YCp50-MYO2*	Yau *et al*., 2014
LSW9506	*MATa, vac17Δ, vac8Δ, myo2Δ::YCP50-MYO2*	Yui Jin
LSW13976	*MATa, vac17Δ, VPH1-mCherry*	Yui Jin
LSW8226	*MATa, YFP-PTS1::LEU2, myo2 delta::TRP1, YCP50-MYO2*	Yui Jin
LSW7656	*MATa, leu2,3-112 ura3-52 his3-Δ200 trp1-Δ901 lys2-801 suc2-Δ9 vac8Δ::VAC8-mRFP-HIS3 vac17Δ::TRP1*	Tang *et al*., 2006

Table S4 Plasmids used in this study

Plasmid name	Description	Reference
pRS416-Vac17	CEN, URA3	Catlett *et al*., 1998
pRS416-Vac17-3xEnvy	CEN, URA3	This study
pRS416-*vac17Δ18-108*-3xEnvy	CEN, URA3	This study
pRS416-*vac17Δ195-250*-3xEnvy	CEN, URA3	This study
pRS416-*vac17Δ110-157*-3xEnvy	CEN, URA3	This study
pRS413-Myo2	CEN, HIS3	Yau *et al*., 2013
pRS413-mCherry-Myo2	CEN, HIS3	Jin *et al*., 2011
pRSF-Duet-1	Kan	Gift from Amir Kahn
pRSF-Strep II-Myo2 Tail(1150-1567)	Kan	This study
pMALc2H10T	Amp	Kristelly *et al*., 2008
pMBP-10xhis-TEV-Vac17(1-157)	Amp	This study
pMBP-10xhis-TEV-Vac17(110-157)	Amp	This study
pMBP-10xhis-TEV-Vac17(1-109)	Amp	This study

Table S5 Cryo-EM Data Collection, Refinement, and Validation Statistics

	Vac17+Myo2 tail complex<br>EMD
**Data collection**	
Grids	UltrAuFoil
Vitrification method	FEI Vitrobot
Automation software	Leginon
Session name	23jun08b
Microscope	Titan Krios G3
Voltage (keV)	300
Recording mode	Counting
Detector	K3
Energy filter slit width	Gatan Bioquantum imaging filter (20 eV)
Magnification	81,000X
Pixel size at detector (Å/pixel)	1.085
Total electron exposure (e–/Å^2)	73.2
Exposure rate (e-/pixel/sec)	21.5
Number of frames	40
Defocus range (μm)	0.8-2.0
**Data processing**	
Software(s) used	CryoSPARC v4
Micrographs collected (no.)	4,605
Micrographs used (no.)	3,224
Particles extracted (no.)	1,387,051
Final particle images (no.)	618,699
Final particles in refinement	144,724 & 31,238
Symmetry	C1
Map resolution (Å)	5.75 & 7.21
FSC 0.5	7.4/6.3/5.8 & 8.1/7.0/7.3
(unmasked/masked/corrected)	-585.7 & -451.8
Map sharpening B factor	
**Refinement**	
Refinement package	Real-space refinement on PHENIX (v1.20.1-4487)
Initial model used	Trimmed Alphafold model of Vac17(127-147) bound Myo2(1151-1569)
**Model composition**	
Non-hydrogen atoms	3,430
Protein residues	423
**Validation**	
MolProbity score	1.74
CaBLAM outliers	0.24
**Ramachandran plot**	
Favored (%)	98.32
Allowed (%)	1.68

Figure S1

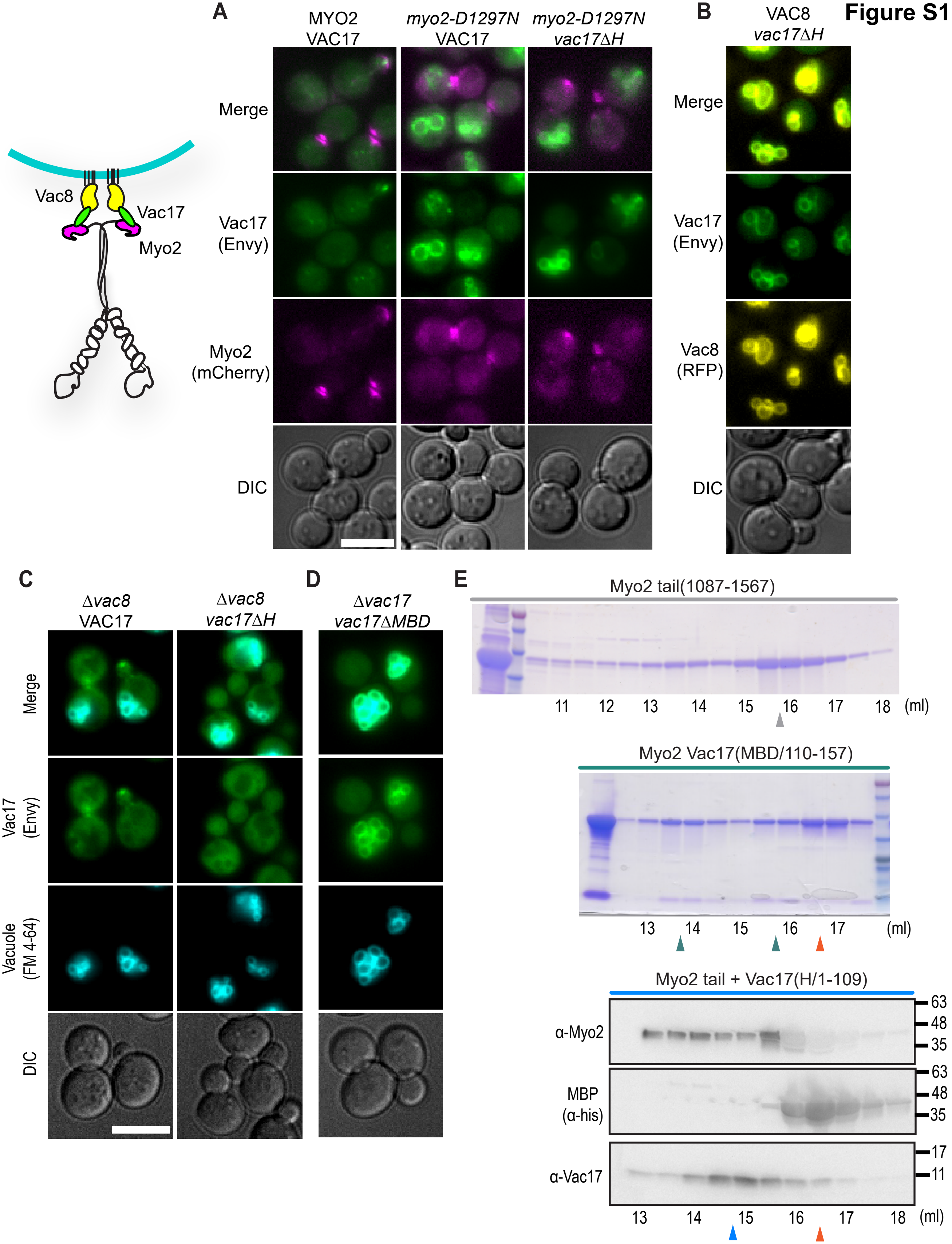

A
MYO2
VAC17
myo2-D1297N
VAC17
myo2-D1297N
vac17ΔH
Merge
Vac17
(Envy)
Myo2
(mCherry)
DIC
Vac8
Vac17
Myo2
B
VAC8
vac17ΔH
Merge
Vac17
(Envy)
Vac8
(RFP)
DIC
C
Δvac8
VAC17
Δvac8
vac17ΔH
Merge
Vac17
(Envy)
Vacuole
(FM 4-64)
DIC
D
Δvac17
vac17ΔMBD
E
Myo2 tail(1087-1567)
11 12 13 14 15 16 17 18 (ml)
Myo2 Vac17(MBD/110-157)
13 14 15 16 17 (ml)
Myo2 tail + Vac17(H/1-109)
α-Myo2
MBP
(α-his)
α-Vac17
63 48 35
63 48 35
17 11
13 14 15 16 17 18 (ml)

Figure S2

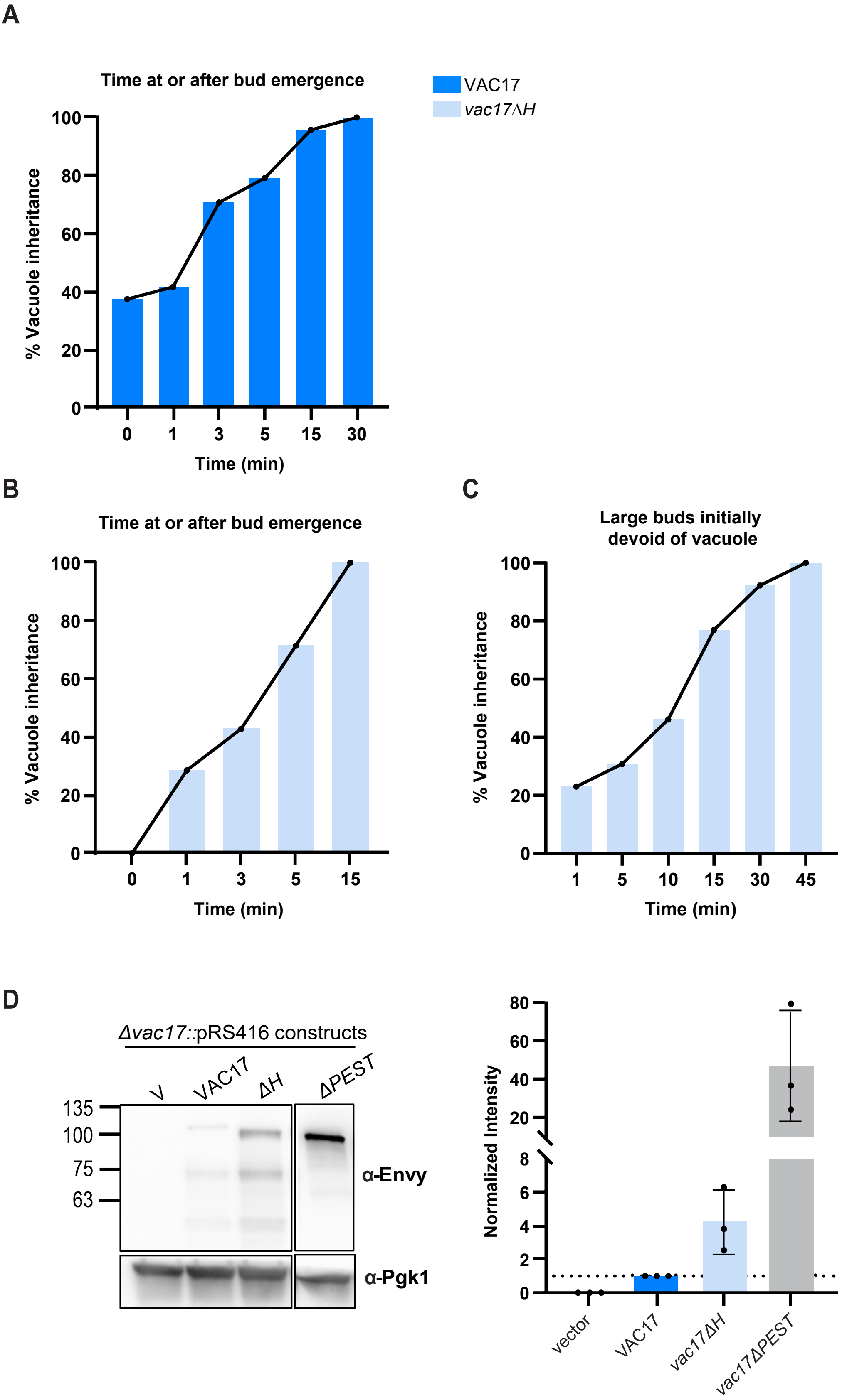
A
Time at or after bud emergence
VAC17
vac17ΔH
% Vacuole inheritance
100
80
60
40
20
0
0
1
3
5
15
30
Time (min)
B
Time at or after bud emergence
% Vacuole inheritance
100
80
60
40
20
0
0
1
3
5
15
Time (min)
C
Large buds initially devoid of vacuole
% Vacuole inheritance
100
80
60
40
20
0
1
5
10
15
30
45
Time (min)
D
Δvac17::pRS416 constructs
V
VAC17
ΔH
ΔPEST
135
100
75
63
α-Envy
α-Pgk1
Normalized Intensity
80
60
40
20
8
6
4
2
0
vector
VAC17
vac17ΔH
vac17ΔPEST

Figure S3

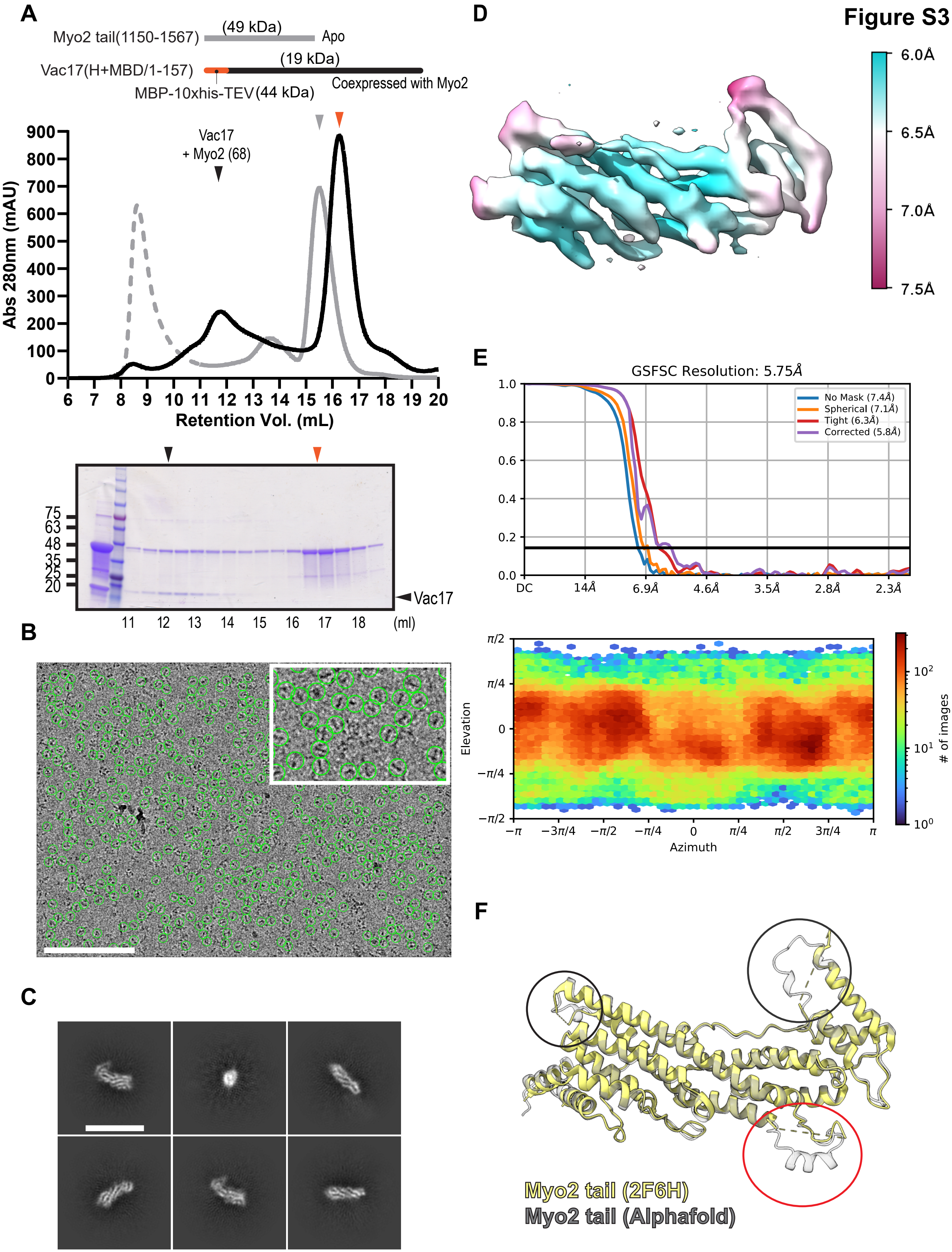
A
Myo2 tail(1150-1567)
(49 kDa)
Apo
Vac17(H+MBD/1-157)
(19 kDa)
MBP-10xhis-TEV(44 kDa)
Coexpressed with Myo2
Vac17
+ Myo2 (68)
Abs 280nm (mAU)
Retention Vol. (mL)
Vac17
(ml)
B
C
D
6.0Å
6.5Å
7.0Å
7.5Å
E
GSFSC Resolution: 5.75Å
No Mask (7.4Å)
Spherical (7.1Å)
Tight (6.3Å)
Corrected (5.8Å)
Elevation
Azimuth
# of images
F
Myo2 tail (2F6H)
Myo2 tail (Alphafold)

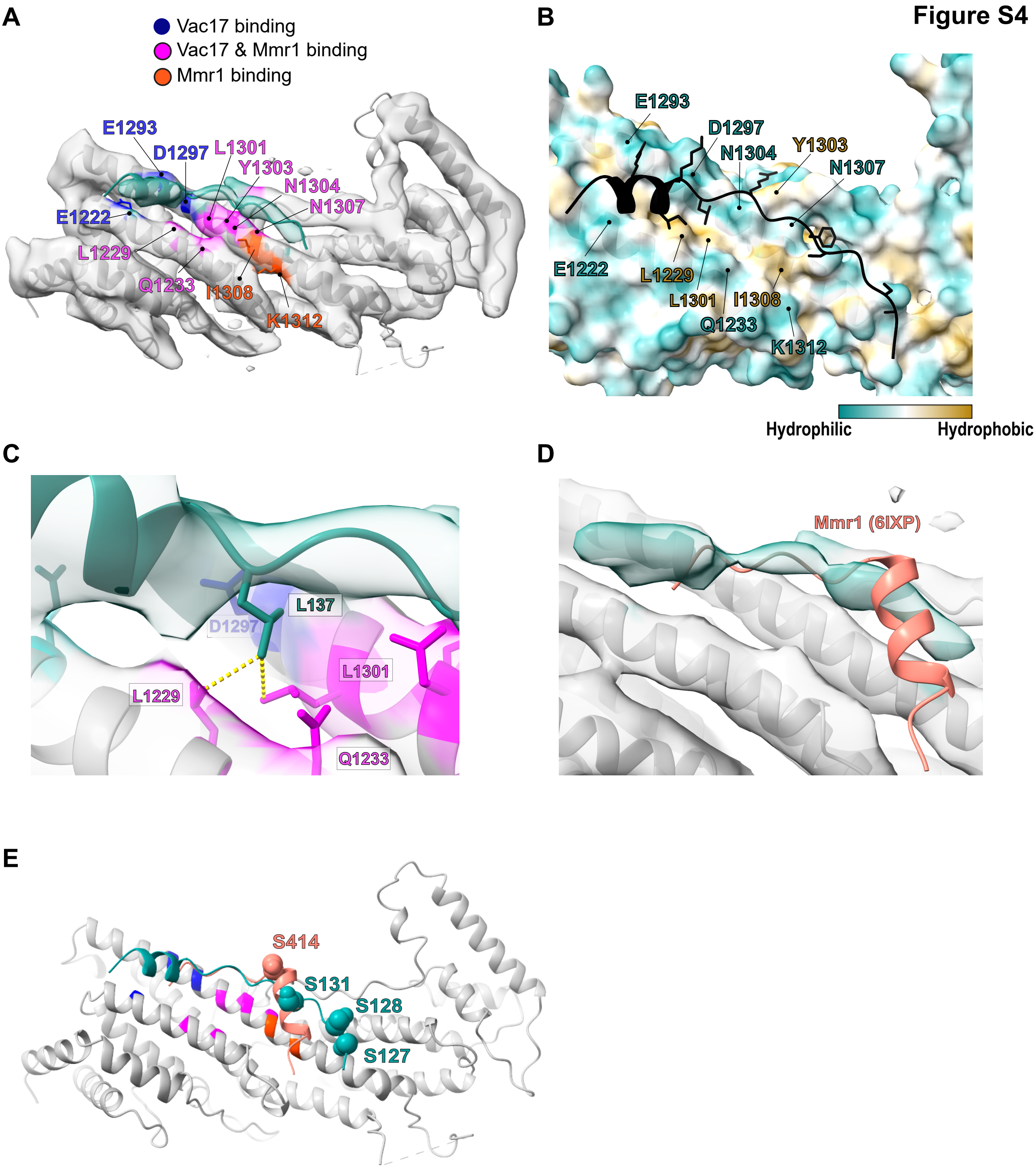
Figure S4
A
Vac17 binding
Vac17 & Mmr1 binding
Mmr1 binding
E1293
D1297
L1301
Y1303
N1304
N1307
E1222
L1229
Q1233
I1308
K1312
B
E1293
D1297
N1304
Y1303
N1307
E1222
L1229
L1301
I1308
Q1233
K1312
Hydrophilic
Hydrophobic
C
L137
D1297
L1301
L1229
Q1233
D
Mmr1 (6IXP)
E
S414
S131
S128
S127

Figure S5

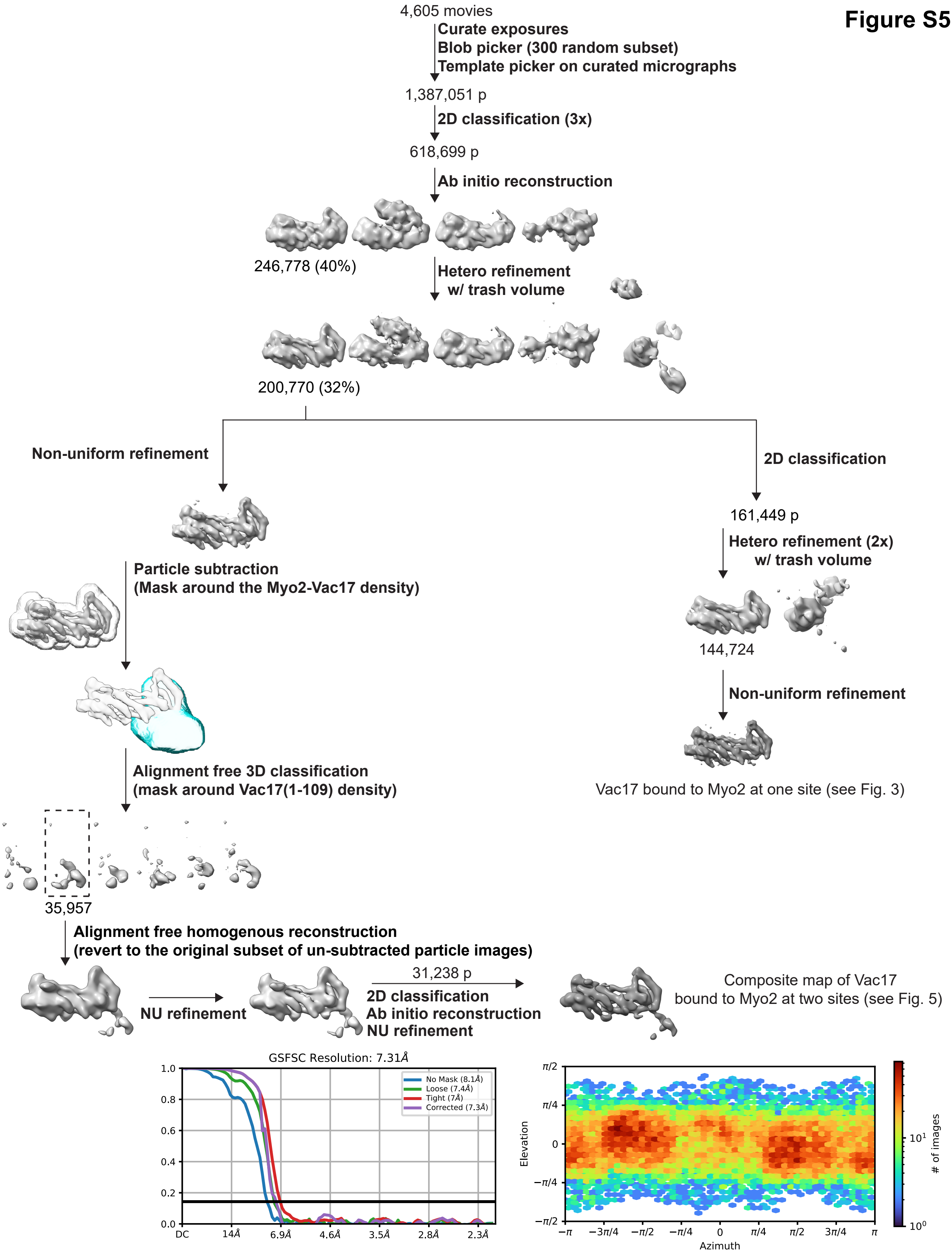
4,605 movies
Curate exposures
Blob picker (300 random subset)
Template picker on curated micrographs
1,387,051 p
2D classification (3x)
618,699 p
Ab initio reconstruction
246,778 (40%)
Hetero refinement
w/ trash volume
200,770 (32%)
Non-uniform refinement
2D classification
161,449 p
Hetero refinement (2x)
w/ trash volume
144,724
Non-uniform refinement
Vac17 bound to Myo2 at one site (see Fig. 3)
Particle subtraction
(Mask around the Myo2-Vac17 density)
Alignment free 3D classification
(mask around Vac17(1-109) density)
35,957
Alignment free homogenous reconstruction
(revert to the original subset of un-subtracted particle images)
NU refinement
31,238 p
2D classification
Ab initio reconstruction
NU refinement
Composite map of Vac17
bound to Myo2 at two sites (see Fig. 5)
GSFSC Resolution: 7.31Å
No Mask (8.1Å)
Loose (7.4Å)
Tight (7Å)
Corrected (7.3Å)
1.0
0.8
0.6
0.4
0.2
0.0
DC
14Å
6.9Å
4.6Å
3.5Å
2.8Å
2.3Å
π/2
π/4
0
−π/4
−π/2
Elevation
−π
−3π/4
−π/2
−π/4
0
π/4
π/2
3π/4
π
Azimuth
# of images
